# Novel Microwave-Assisted Synthesis of the Immunomodulator Organotellurium Compound Ammonium Trichloro(dioxoethylene-*O*,*O*′)tellurate (AS101)

**DOI:** 10.3390/ijms15023287

**Published:** 2014-02-21

**Authors:** M. Pilar Vázquez-Tato, Alberto Mena-Menéndez, Xesús Feás, Julio A. Seijas

**Affiliations:** Department of Organic Chemistry, Faculty of Science, University of Santiago de Compostela, E-27002 Lugo, Spain; E-Mails: pilar.vazquez.tato@usc.es (M.P.V.-T.); menamenendez@hotmail.es (A.M.-M.); xesus.feas@usc.es (X.F.)

**Keywords:** Tellurium, AS101, ammonium trichloro[1,2-ethanediolato-*O,O*′]-tellurate, microwave irradiation, antitumoral

## Abstract

Ammonium trichloro[1,2-ethanediolato-*O*,*O*′]-tellurate (AS101) is the most important synthetic Te compound from the standpoint of its biological activity. It is a potent immunomodulator with a variety of potential therapeutic applications and antitumoral action in several preclinical and clinical studies. An experimental design has been used to develop and optimize a novel microwave-assisted synthesis (MAOS) of the AS101. In comparison to the results observed in the literature, refluxing Te(IV) chloride and ethylene glycol in acetonitrile (Method A), or by refluxing Te(IV) chloride and ammonium chloride in ethylene glycol (Method B), it was found that the developed methods in the present work are an effective alternative, because although performance slightly decreases compared to conventional procedures (75% *vs.* 79% by Method A, and 45% *vs.* 51% by Method B), reaction times decreased from 4 h to 30 min and from 4 h to 10 min, by Methods A and B respectively. MAOS is proving to be of value in the rapid synthesis of compounds with new and improved biological activities, specially based on the benefit of its shorter reaction times.

## 1. Introduction

Tellurium (Te), discovered in 1783, is one of the least abundant elements in the earth’s lithosphere and it is one of the five elements that has never been reported in sea water. However, it is the fourth most abundant trace element in the human body, and is unusually abundant in human food [1]. The investigation of therapeutic activities of Te compounds is rather limited in literature, despite the relative abundance of Te in the human body [2–4]. The similarity of selenium and Te encompasses not only their names and positions on the periodic table but, to a large degree, their chemistry and biochemistry. Also, similar to Se 40 years ago, it is possible that the toxic element Te will ultimately be found to be an essential element [5].

Te chemistry has made great progress in the last few years [6]. Obtention of inorganic and organic Te compounds, as well as their use as reagents to perform specific organic manipulations and synthesis, have been well reviewed in literature, demonstrating Te compounds as a powerful tool in a broad range of organic chemical manipulations, often characterized by their selective behaviour [7,8].

Ammonium trichloro[1,2-ethanediolato-*O*,*O*′]-tellurate (AS101) is the most important synthetic Te compound from the standpoint of its biological activity. It is a potent immunomodulator with a variety of potential therapeutic applications and antitumoral action presently being investigated in several preclinical and clinical studies [9]. AS101 has also been shown to possess antibacterial ability [10,11], antioxidative properties [12], mediates anti-inflammatory and anti-apoptotic effects [13,14], protects from chemotherapy-induced bone marrow toxicity and alopecia [15], restores dopaminergic neurons in Parkinson’s disease models [16] and may be useful as a treatment for type 2 diabetes [17], multiple sclerosis [18], premature ovarian failure in cancer treatments [19], atopic dermatitis [20] and in clinical immunosuppression conditions involving AIDS [21] and West Nile virus [22].

Over recent years, heating and driving chemical reactions by microwave (MW) energy has been a significant interest in the scientific community, in particular applied to microwave-assisted organic synthesis (MAOS), medicinal chemistry, drug discovery and polymer science. Some excellent reviews and books are present in the literature [23–32]. In summary, the capacity of microwaves to couple energy directly to the material is the primary advantage of MW processing as compared to conventional techniques, allowing shorter reaction times, uniform heating, higher yields and often enhancing material properties and product purities by reducing unwanted side reactions. Moreover, the combination of solid supported reagents and scavengers, polymer supported reactions, solvent free conditions and MAOS methodologies, has several advantages in the ecofriendly approach termed green chemistry [33–36].

MAOS is proving to be instrumental in the rapid synthesis of compounds with new and improved biological activities. The extensive use of MW irradiation for the synthesis of molecules of pharmacological interest has contributed to improve the access to different chemical scaffolds by applying new methodologies and techniques, specially based on the benefits related to better yields and shorter reaction times. Clearly MW irradiation is becoming a fundamental tool for optimizing key steps in the synthesis of target compounds within the field of drug discovery [23,33,34,37,38].

Continuing with our studies on MAOS [39–43], we draw attention to the AS101 compound. The main objective of the present paper was to develop an optimized a method for MW assisted synthesis of AS101. To achieve this objective, independent variables such as MW power, pressure, synthesis temperature and time of the reaction on AS101 obtention were examined.

## 2. Results and Discussion

**Scheme 1.**
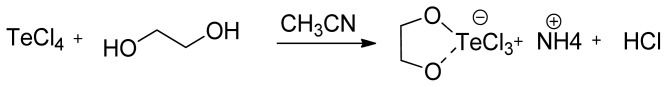
Synthesis of AS101.

AS101 was previously synthesized by refluxing Te(IV) chloride and ethylene glycol in acetonitrile as shown in Scheme 1 (Method A), or by refluxing Te(IV) chloride and ammonium chloride in ethylene glycol (Method B) [44]. A number of very interesting syntheses have been performed omitting the solvent from the reaction and a majority of the publications contain work conducted in this manner. Further, the solventless microwave-assisted reaction is now gaining popularity as it provides an opportunity to work with open vessels, thus avoiding the risk of high pressure development and with a possibility of upscaling the reactions on preparative scale [27,45]. As a first approach, the reactants were irradiated at 120 °C under solvent-free conditions. However, only decomposition of the reaction mixture was achieved. Thus, the reactions (Method A and Method B) were checked in the presence of solvent.

Both methods have been optimized using experimental designs: Plackett–Burman (PB) and central composite (CC). PB allows the unbiased estimation of all the main effects for all variables, requiring few experiments. The examined factors and their levels are presented in Table 1. PB requires that the frequency of each level of a variable should be equal and that in each test the number of high and low variables should also be equal. Then, the effects of changing the other variables cancel each other out, while determining the effect of any particular variable.

Although MAOS and its applications have undergone rapid growth over the last decade, the technology is not yet employed routinely in all synthetic laboratories. A significant obstacle to implementation concerns the empirical work required to adapt established conditions into alternatives. However, experimental designs and statistical analysis of a designed set of experiments allows for much more data to be obtained than is normally the case with the one variable at a time approach [46].

Table 2 summarizes the design matrix and the AS101 yield obtained in each of the experiments; the results are expressed as percentages. Experiments based on Method A, displayed the best AS101 yielding rates (53.00% ± 1.4% to 78.5% ± 0.7%) in comparison with Method B (20.00% ± 4.2% to 45.00% ± 0.3%).

**Figure 1.**
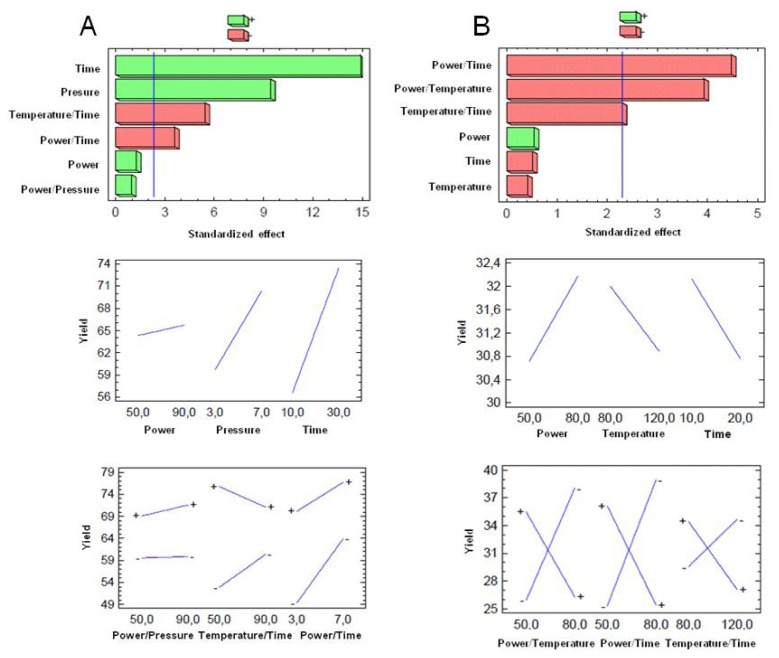
Standardised (*p* = 95%) main effects Pareto charts for the Plackett–Burman desing for the different variables studied and graphs of main effects in AS101 yields by Method A (**A**) and by Method B (**B**).

The numerical analysis of the results given in the recovery column of Table 2, were evaluated by an ANOVA test (Table 3) and the effects were visualized using the Pareto chart shown in Figure 1.

On Pareto charts, the bar length is proportional to the absolute value of the estimated main effect and a vertical reference line corresponding to 95% confidence interval is included. An effect is significant if it exceeds this reference line while a positive or negative sign means that the response is enhanced or reduced, respectively.

Equations (1) and (2) describing the empirical relationship between the independent variables and response were generated and are given underneath:

(1) $$\begin{array}{c} \text{AS}101\;(\text{Yield in }\%\;\text{by Method A})=5.83703+0.272984\;X_{1\text{a}}+3.70453\;X_{2\text{a}}+2.41162\\ X_{3\text{a}}+0.0136094\;X_{1\text{a}}X_{2\text{a}}-0.0152844\;X_{1\text{a}}X_{3\text{a}}-0.100406\;X_{2\text{a}}X_{3\text{a}} \end{array}$$

(2) $$\begin{array}{c} \text{AS}101\;(\text{Yield in }\%\;\text{by Method B})=-208.317+3.04337\;X_{1\text{b}}+1.59551\;X_{2\text{b}}+8.25513\\ X_{3\text{b}}-0.0177771\;X_{1\text{b}}X_{2\text{b}}-0.081125\;X_{1\text{b}}X_{3\text{b}}-0.0311937\;X_{2\text{b}}X_{3\text{b}} \end{array}$$

The magnitude and direction of the factor coefficient in the generated equations explains the nature of the effect of factors on the AS101 yield. The *R*^2^ values obtained were 0.9779 and 0.8394 for methods A and B respectively. *R*^2^ value gives a measure of how much variability in the observed response can be explained by the experimental parameters and their interactions. When expressed as a percentage, it implies that a total variation of 97.79% in AS101 yield obtained by Method A can be attributed to the independent variables and only 2.20% cannot be ascribed to them. The predicted *R*^2^ values are in acceptable agreement with the adjusted *R*^2^ of 0.9632 and 0.7324 for Methods A and B respectively.

The ANOVA results are given in Table 3. The *p* value serves as a tool for checking the significance of each of the coefficients and is indicative of the interaction strength of each independent variable. Low values of *p* < 0.05 indicate high significance of the corresponding coefficients.

**Figure 2.**
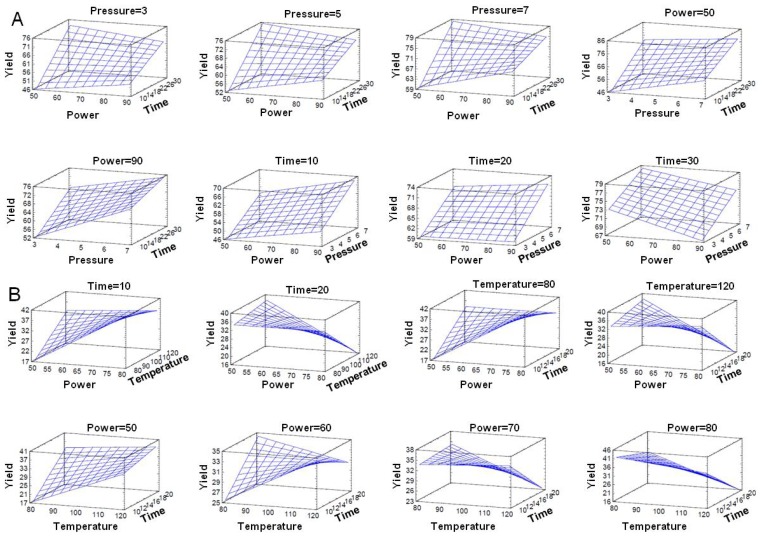
Response-surface graphs representing: the effect of power, pressure and time on the AS101 yield by Method A (**A**) and relative effect of time; temperature and power on the AS101 yield by Method B (**B**).

A response surface methodology (RSM) using a central composite were generated by varying the levels of two factors while keeping the third one constant (Figure 2). Pressure, time and power were choosen as the base factors with increments of 0.2 bar, 1 min and 1 watt respectively.

For Method A, according to the design of experiments and the results obtained, it was determined that the optimum reaction conditions were 50 watts, 7 bar and 30 min. Subsequent experiments were expanded and the pressure time up to 9 bar and 50 min but previously obtained results were not improved. Reactions were also performed in three stages of 10 min each, removing the HCl produced in the reaction to improve the yield with no positive results. As variables that positively affect the reaction are the pressure and time, the combined effect is negative for the reaction, but significantly lower than the positive effect that both generate. In relation to results observed in the literature, as [44] observed a 4 h and 30 min reaction with a yield of 75% to 79%, it can therefore be concluded that the MAOS of AS101 using Method A is more effective compared to alternative conventional procedure.

For Method B, the optimum reaction conditions were 80 watts, 80 °C and 10 min of reaction. Further experiments were made to increase the power to 120 watts without improving the results previously obtained. Likewise AS101 synthesis performed using Method B with the experiment in a closed tube, did not provide satisfactory results. The reaction did not start to occur until after 2–3 min, at which time the pressure began to increase and control was lost. Power is the variable that positively affects the reaction, but negligible weight was evident, also in subsequent experiments. We can conclude therefore that this method does not fit as well to the statistical parameters studied as Method A. It is not as reproducible and generates a lot of noise in the system. In relation to results observed in the literature, as [44] found a 4 h and 10 min reaction with a yield of 51% to 45%, it can be concluded that the MAOS of AS101 by Method B is more effective, because although performance decreases compared to the conventional procedure by 6%, it took 95% less time.

## 3. Experimental Section

### 3.1. General Experimental Procedure

All the chemical reagents used were purchased from Sigma Chemical Co. (St. Louis, MO, USA) and were of analytical grade. MAOS was performed in an Emrys Creator^®^ single-mode microwave cavity producing controlled irradiation at 2.45 GHz (Biotage AB, Uppsala, Sweden). The reaction times refer to the hold times at the temperature indicated. The temperature was monitored with an IR sensor equipped on the outside of the reaction vessel. Two procedures for AS101 obtention were performed. Samples of AS101 are available from the authors.

#### 3.1.1. AS101 Obtention by Method A

TeCl_4_ (0.675 g) and ethylene glycol (0.387 g) were dissolved in acetonitrile (5 mL). The mixture was MW irradiated for 30 min at 50 W. On cooling the reaction mixture, a white crystalline solid is obtained, washed with acetonitrile, filtered and vacuum dried. The solid (615 mg, 79%) was identified as:

**Found** (%): C, 7.70; H, 2.58; N, 4.49. C_2_H_8_Cl_3_NO_2_Te.
**Calculated** (%): C, 7.85; H, 2.37; N, 4.42.
**IR** (***Golden-Gate***): v = 3183 (NH): 1390 (NH_4_^+^): 1019: 894 cm^−1^.
**NMR-^1^H** (**DMSO-*d****_6_*, **δ**, **ppm**): 4.36 (s, 4H, CH_2_): 7.16 (t, 4H, *J* = 50 Hz, NH_4_^+^).
NMR-^13^C (DMSO-*d_6_*, δ, ppm): 68.13.
**EM** (*m*/*z*): 292 (M^+^-NH_4_,1), 290 (M^+^–NH_4_^+^,1), 260 (M^+^–NH_4_^+^–Cl [Te_130_, 2Cl_35_],7), 258 (M^+^–NH_4_^+^–Cl [Te_128_, 2Cl_35_],13), 256 (M^+^–NH_4_^+^–Cl [Te_126_, 2Cl_35_],13), 254 (9), 224 (42), 223 (35), 221 (19), 200 (36), 198 (27), 196 (14), 190 (69), 188 (63), 186 (37), 165 (21), 163 (18), 161 (11), 146 (5), 130 (25), 128 (23), 126 (13).

#### 3.1.2. AS101 Obtention by Method B

TeCl_4_ (1.35 g) and NH_4_Cl (0.387 g) were dissolved in ethylene glycol (5 mL). The mixture was MW irradiated for 10 min at 80 W. On cooling the reaction mixture, a white crystalline solid is obtained, washed with acetonitrile, filtered and vacuum dried. The solid (706 mg, 45%) was identified as:

**Found** (%): C, 7.81; H, 2.33; N, 4.45. C_2_H_8_Cl_3_NO_2_Te.
**Calculated** (%): C, 7.70; H, 2.58; N, 4.49.
**IR** (***Golden-Gate***): 3198 (NH): 1399 (NH_4_^+^): 1017: 889 cm^−1^.
**NMR-^1^H** (**DMSO-*d****_6_*, **δ**, **ppm**): 4.36 (s, 4H, CH_2_): 7.16 (s, 4H, NH_4_^+^).
NMR-^13^C (DMSO-*d_6_*, δ, ppm): 68.10.
**EM** (*m*/*z*): 292 (M^+^–NH_4_^+^,1), 290 (M^+^–NH_4_^+^,1), 260 (M^+^–NH_4_^+^–Cl [Te_130_,2Cl_35_], 11), 258 (M^+^–NH_4_^+^–Cl [Te_128_, 2Cl_35_],20), 256 (M^+^–NH_4_^+^–Cl [Te_126_, 2Cl_35_],21), 254 (14), 224 (52), 223 (43), 221 ( 23), 200 (46), 198 (36), 196 (18), 190 (100), 188 (92), 186 (54), 165 (27), 163 (23), 161 (14), 146 (7), 130 (32), 128 (30), 126 (18).

### 3.2. ^1^H-NMR and ^13^C-NMR Analysis

^1^H-NMR and ^13^C-NMR analyses were performed on Varian Mercury 300 (300 MHz for 1H) instrument (Agilent Technologies^®^, Palo Alto, CA, USA), equipped with a 5 mm probe. Each obtained product, was dissolved in 400 μL of DMSO-*d_6_*, (Sigma-Aldrich^®^, Madrid, Spain) shaken in a vortex mixer, and the resulting mixture was placed into a 5-mm diameter ultra-precision NMR sample tubes (Norell^®^, Landisville, PA, USA). The temperature of the sample in the probe was 30 °C. The chemical shifts are reported in ppm, using the solvent proton signal as standard. The area of the signals was determined by using the equipment software, and the integrations were carried out three times to obtain average values. All figures of the ^1^H-NMR spectra plotted at a fixed value of absolute intensity to be valid for comparative purposes. ^13^C-NMR analysis was performed at 189 MHz for ^13^C. The same samples subjected to ^1^H-NMR were used for ^13^C-NMR.

### 3.3. MS Analysis

A Finnigan™ TRACE™ DSQ™ (Thermo Electron Corporation, Austin, TX, USA) quadrupole mass spectrometer and a direct sample probe fitted with a direct exposure probe was used for sample introduction and analysis. Mass spectra were recorded in electron impact (EI) mode at an ionization voltage of 70 eV. Data were collected and processed by Xcalibur™ software package (Thermo Finnigan, Austin, TX, USA).

### 3.4. Infrared Spectroscopy (FTIR)

Spectra were recorded using an ABB Bomem MB 102 spectrometer (ABB-Bomem Inc., Quebec City, QC, Canada). By means of a single reflection diamond ATR accessory (Specac Inc.: Swedesboro, NJ, USA). The samples were previously dried overnight under reduced pressure and homogenized by grinding. The frequency value of each band was obtained automatically by the software.

### 3.5. Optimization of MAOS AS101 Procedure

A Plackett–Burman (PB) design was used to evaluate the influence of factors involved in the MAOS of AS101 procedure in a reduced number of runs for the two methods (A and B) considered in this study. The total number of experiments to be carried out is *K* + 1, where *K* is the number of variables. Each variable is represented at two levels, high and low denoted by (+) and (−) respectively. The main effect of each variable on AS101 yield was calculated as the difference between the average of measurements made at high (+1) and low (−1) levels setting, by using the following equation:

(3) $$E_{\left(Xi\right)}=\sum Y_{(+)i}-\sum Y_{(-)i}/N$$

*Y*_(+)_*_i_* and *Y*_(−)_*_i_* are the AS101 yield from the experimental runs in which the variables being tested are considered at their maximum and minimum levels respectively and *N* is the half number of experiments carried out. When *E*_(_*_Xi_*_)_ is positive, the influence of the variable is greater at the high concentration, and when it is negative, the influence of the variable is greater at the low concentration. Subsequently, a composite central design was set up for the optimization of significant experimental factors, analyzing the individual response and then applying the above mentioned desirability function. The obtained models of the regression were validated and analyzed using the analysis of variance (ANOVA).

### 3.6. Statistical Analyses

For experimental design modelling, programs from Statgraphics Plus 4.0 routine (Statgraphics Graphics Corporation, STSC, Rockville, MD, USA) were used.

## 4. Conclusions

In summary, microwave-assisted organic synthesis has proven to be a useful tool for the obtention of the immunomodulator organotellurium compound, ammonium trichloro(dioxoethylene-*O*,*O*′)tellurate (AS101). Two easy, efficient and fast procedures for syntheses of AS101 were developed since the reaction took place in only 30 min with a yield of 79% (Method A) and 10 min with a yield of 45% (Method B); and by conventional heating, 4 h are necessary with a 75% yield (Method A) and 51% yield (Method B). We believe that the proposed procedure has the potential to evolve to incorporate new processes for obtaining AS101 derivatives, since the optimization of newly discovered lead compounds relies upon the advancement of synthetic technologies.

## Figures and Tables

**Table 1.** Factor levels in the Plackett–Burman design for MAOS AS101 synthesis.

	Key	High level (+)	Low level (−)	Unit
Method A				
Power	*X*_1a_	90	50	watt
Pressure	*X*_2a_	7	3	bar
Time	*X*_3a_	30	10	minute
Method B				
Power	*X*_1b_	80	50	watt
Temperature	*X*_2b_	120	80	°C
Time	*X*_3b_	20	10	minute

**Table 2.** Design matrix and response values in Plackett–Burman factorial design for MAOS AS101 synthesis.

	*X*_1a_	*X*_2a_	*X*_3a_	*X*_1b_	*X*_2b_	*X*_3b_	Yield Method A	Yield Method B
1	+	+	+	+	−	−	73.5 ± 2.1	20.0 ± 4.2
2	−	+	+	−	+	−	78.5 ± 0.7	34.5 ± 2.1
3	−	−	+	+	−	+	67.0 ± 2.8	38.0 ± 5.7
4	+	−	−	+	+	−	53.0 ± 1.4	45.0 ± 0.3
5	−	+	−	+	+	+	78.5 ± 0.7	36.5 ± 0.7
6	+	−	+	−	+	+	75.5 ± 0.7	31.0 ± 2.8
7	+	+	−	−	−	+	53.0 ± 1.4	33.0 ± 1.4
8	−	−	−	−	−	−	0	0

Method A: *X*_1a_ (power), *X*_2a_ (pressure) and *X*_3a_ (time); Method B: *X*_1b_ (power), *X*_2b_ (temperature) and *X*_3b_ (time).

**Table 3.** Design matrix and response values in Plackett–Burman factorial design.

Source	Sum of squares	d*f*	Mean square	*F* value	*p* value Prob > *F*
*X*_1a_	7.99	1	7.99	1.57	0.2452
*X*_2a_	449.122	1	449.122	88.36	<0.0001
*X*_3a_	1128.12	1	1128.12	221.94	<0.0001
*X*_1a_* *X*_2a_	4.74	1	4.74	0.93	0.3624
*X*_1a_* *X*_3a_	149.51	1	149.51	29.41	<0.0001
*X*_2a_* *X*_3a_	64.52	1	64.52	12.69	0.0074

Total error	40.66	8	5.08		
Cor total	1846.32	15			

*X*_1b_	8.57	1	8.57	0.29	0.6048
*X*_2b_	4.98	1	4.98	0.17	0.6921
*X*_3b_	7.54	1	7.54	0.26	0.6269
*X*_1b_* *X*_2b_	455.07	1	455.07	15.40	0.0044
*X*_1b_* *X*_3b_	592.31	1	592.31	20.05	0.0021
*X*_2b_* *X*_3b_	155.68	1	155.68	5.27	0.0505

Total error	236.35	8	29.54		
Cor total	1472.22	15			

Method A: *X*_1a_ (power), *X*_2a_ (pressure) and *X*_3a_ (time); Method B: *X*_1b_ (power), *X*_2b_ (temperature) and *X*_3b_ (time).
